# Hospital Notes

**Published:** 1902-07

**Authors:** 


### HOSPITAL NOTES.

Announcement is made by Dr. Nelson W. Wilson, and Dr. James A. Gardner, attending surgeons in the department of kidney and genitourinary diseases at the Emergency Hospital, corner of Pine and Eagle Streets, of the completion of the work of equipping the genitourinary dispensary. Patients who are unable to pay for treatment are received each day at 11.30. Dr. Gardner has Monday, Wednesday and Friday; Dr. Wilson, Tuesday, Thursday and Saturday.

---

A new dispensary will be opened about July 1, 1902, by Dr. Lillian Craig Randall, of the Riverside Hospital, who has been granted a license by the state board of charities to open an out-patient department of her hospital. It is to be located on the ground floor of the Fitch Institute. The department will include a dispensary to the poor and the care of emergency cases. One branch of the institution will be a department for the treatment of tuberculosis. A trained nurse will be in attendance to care for emergency and other cases. Dr. Randall already has a handsome ambulance of modern make and equipment, which will be used for the new dispensary.

The following-named physician will serve on the medical staff of the institution: Henry D. Ingraham, John C. Thompson, George F. Cott, H. C. Rooth, J. H. Potter, Grover W. Wende, Charles E. Brownell, James E. King, James Croff, Carro Cummings Croff, Thomas H. McKee, James A. Gibson, Jane N. Frear and Mary M. Huntley.

